# Waste Rose Flower and Lavender Straw Biomass—An Innovative Lignocellulose Feedstock for Mycelium Bio-Materials Development Using Newly Isolated *Ganoderma resinaceum* GA1M

**DOI:** 10.3390/jof7100866

**Published:** 2021-10-15

**Authors:** Galena Angelova, Mariya Brazkova, Petya Stefanova, Denica Blazheva, Veselin Vladev, Nadejda Petkova, Anton Slavov, Petko Denev, Daniela Karashanova, Roumiana Zaharieva, Atanas Enev, Albert Krastanov

**Affiliations:** 1 Department of Biotechnology, University of Food Technology, 26 Maritsa Blvd., 4002 Plovdiv, Bulgaria; g_angelova@uft-plovdiv.bg (G.A.); petyastefanova@uft-plovdiv.bg (P.S.); a_krastanov@uft-plovdiv.bg (A.K.); 2 Department of Microbiology, University of Food Technology, 26 Maritsa Blvd., 4002 Plovdiv, Bulgaria; d_blazheva@uft-plovdiv.bg; 3 Department of Mathematics, Physics and Information Technologies, Faculty of Economics, University of Food Technologies, 26 Maritsa Blvd., 4002 Plovdiv, Bulgaria; v.p.vladev@abv.bg; 4 Department of Organic and Inorganic Chemistry, University of Food Technologies, 26 Maritsa Blvd., 4002 Plovdiv, Bulgaria; petkovanadejda@abv.bg (N.P.); antons@uni-plovdiv.net (A.S.); 5 Laboratory of Biologically Active Substances, Institute of Organic Chemistry with Centre of Phytochemistry, Bulgarian Academy of Sciences, 139 Ruski Blvd., 4000 Plovdiv, Bulgaria; petkodenev@yahoo.com; 6 Institute of Optical Materials and Technologies, Bulgarian Academy of Sciences, Acad. Georgy Bonchev Str., 1113 Sofia, Bulgaria; dkarashanova@yahoo.com; 7 Department of Building Materials and Insulation, Faculty of Structural Engineering, University of Architecture, Civil Engineering and Geodesy, 1046 Sofia, Bulgaria; zaharieva_fce@uacg.bg; 8 Byomic Ltd., 1000 Sofia, Bulgaria; atanas.enev@biomyc.eu

**Keywords:** hexane extracted rose flowers, steam distilled lavender straw, mycelium bio*-*composites, *Ganoderma resinaceum*, apparent density, water absorbance, compressive resistance

## Abstract

In this study, for the first time, the potential of rose flowers and lavender straw waste biomass was studied as feeding lignocellulose substrates for the cultivation of newly isolated in Bulgaria *Ganoderma resinaceum* GA1M with the objective of obtaining mycelium*-*based bio*-*composites. The chemical characterization and Fourier Transform Infrared (FTIR) spectroscopy established that the proximate composition of steam distilled lavender straw (SDLS) and hexane extracted rose flowers (HERF) was a serious prerequisite supporting the self*-*growth of mycelium bio*-*materials with improved antibacterial and aromatic properties. The basic physico*-*mechanical properties of the developed bio*-*composites were determined. The apparent density of the mycelium HERF*-*based bio*-*composites (462 kg/m^3^) was higher than that of the SDLS*-*based bio*-*composite (347 kg/m^3^) and both were much denser than expanded polystyren (EPS), lighter than medium*-*density fiber board (MDF) and oriented strand board (OSB) and similar to hempcrete. The preliminary testing of their compressive behavior revealed that the compressive resistance of SDLS*-*based bio*-*composite was 718 kPa, while for HERF*-*based bio*-*composite it was 1029 kPa and both values are similar to the compressive strength of hempcrete with similar apparent density. Water absorbance analysis showed, that both mycelium HERF*-* and SDLS*-*based bio*-*composites were hydrophilic and further investigations are needed to limit the hydrophilicity of the lignocellulose fibers, to tune the density and to improve compressive resistance.

## 1. Introduction

Roses and lavender are plant species, which have been highly appreciated for centuries, and because of their diverse biologically active substance content, they have an industrial impact on essential oil production worldwide [1,2]. Bulgaria and Turkey have long traditions in rose oil production and dominate the world market, followed by Iran, Morocco, France, Italy, India and China [1]. Bulgarian production satisfies 40% of the global demand for rose oil. In recent years a drastic increase in lavender cultivation and lavender oil yield is being observed in Bulgaria, which defines its leading position as a lavender oil producer, leaving France behind.

The most common methods for the production of essential oils are water*-*steam distillation, steam distillation and solvent (non*-*polar solvents and liquefied gases) extraction of the raw plant materials. Due to the relatively low concentration of essential oils in the crops, large amounts of plant material are treated and, respectively, a huge quantity of solid residual biomass is generated. More than 20,000 t of steam distilled lavender straw (SDLS), about 29,000 t of steam distilled rose flowers (SDRF) and hexane extracted rose flowers (HERF) are generated each year only in Bulgaria [1].

Generally, rose flower and lavender straw by*-*products are considered waste material and still, most of them are incinerated for energy recovery or discarded near the distilleries, which poses an environmental hazard. This waste biomass is referred to as lignocellulose waste, because of its high content of lignocellulose and hemicellulose compounds, pectic carbohydrates and polyphenols [1,3]. The valuable biological content of HERF and SDLS can be used in innovative “self*-*growing” technologies for obtaining value*-*added materials with sustainable social and economic impact.

In recent years, various lignocellulose wastes with agricultural and forestry origin are studied and used as substrates for the production of sustainable, renewable, biodegradable and eco*-*friendly natural*-*based bio*-*materials for indoor and outdoor applications [4]. The use of biological agents for binding the lignocellulose fibers and turning them into 100% natural and bio*-*recyclable bio*-*materials is the most environmentally friendly mechanism and it revolutionizes the traditional ideas for materials production. “Materials that are grown are better than manufactured” is an innovative promising approach in materials science, aiming to achieve unique self*-*grown material, which has attracted considerable research efforts in recent years [5,6,7,8,9,10,11].

Currently, numerous research teams explore the effect of various combinations between plant lignocellulose substrates and higher fungi to develop functional mycelium*-*based bio*-*composite materials with the potential to replace synthetic materials. Many studies have reported the ability of mushrooms, belonging to the *Trametes*, *Ganoderma* and *Pleurotus* genera, to be cultivated on a range of lignocellulose materials, including various types of cotton seed hulls, corn cobs, peanut shells, cotton from the textile industry, coffee pulp, paper, leaves, coconut powder [6,8,9,12,13,14,15,16,17,18,19,20,21] as well as low*-*quality organic waste streams like saw dust and straw and turning them into mycelium bio*-*composites [22]. The structural and macroscopic characteristics of the mycelium*-*based bio*-*composites are strongly dependent on the fungal species, the type and chemical composition of the substrates and both cultivation conditions and post*-*cultivation processing define their non*-*structural, semi*-*structural or specific applications [4,6,7,11,12,18,22,23,24,25,26]. Due to its fibrous structure and polymeric composition, the mycelium can serve as a matrix and the lignocellulose fibers as reinforcement for the newly obtained mycelium bio*-*material [6,7,13]. The fungal mycelium possesses the advantage of strength and durability performance and together with its non*-*toxicity, fire*-*resistance and hydrophobic capabilities show great potential as a binding element in mycelium bio*-*composites development [8,13,27].

The mycelium*-*based bio*-*composites are completely natural and can be composted after the end of their cycle of use, which would help the transition to a circular economy, keeping the value of materials and resources in the economy as long as possible and generating minimal waste [6,8,22]. They also have the potential to contribute to the new “green” economy by replacing many oil*-*based products and converting lignocellulose waste into value*-*added biodegradable products, which don‘t damage the ecosystem cycles. The lower water, energy and CO_2_ requirements, together with the low environmental impact in comparison with the conventional industries, are the key advantages for the application of self*-*grown bio*-*composites [7,27,28].

The analysis of reference literature revealed that there are many studies showing the potential of waste rose flower and lavender straw biomass as raw materials for aroma and polysaccharides extraction, for recovery of biologically active substances and their application in the food industry and medicine [1,29]. Lavender distilled straw separately or in combination with other lignocellulose substrates was studied by Ratiarisoa et al. [30] and was successfully recycled as a bio*-*aggregate for building materials using metakaolin as a binder.

At present, there are no reports on the usage of rose and lavender industrial processing by*-*products as feeding substrates for mushroom growth aiming at the obtaining of mycelium*-*based bio*-*materials, although their chemical composition suggests they are fully compatible with fungal growth. Lavender and lavandin distilled straw was successfully used as a feeding substrate for *Pycnoporus cinnabarinus* cultivation by Lesage*-*Meessen et al. [29], but for lignocellulose*-*acting enzyme synthesis.

The focus of this study is to explore the chemical characteristics of hexane extracted rose flowers and steam distilled lavender straw, which are a significant waste of the essential oil*-*processing industry and to reveal their realistic potential to be used as feedstock, promoting vegetative fungal growth. The ability of a newly isolated in Bulgaria fungal strain to grow on HERF and SDLS and convert them into mycelium*-*based bio*-*materials was studied. The assessment of the basic physical and mechanical properties of these mycelium*-*based bio*-*materials opens possibilities for further in*-*depth investigations and the production of mycelium HERF*-* and SDLS*-*based bio*-*materials for various applications.

## 2. Materials and Methods

### 2.1. Materials

#### 2.1.1. Substrates

**Figure 1:**
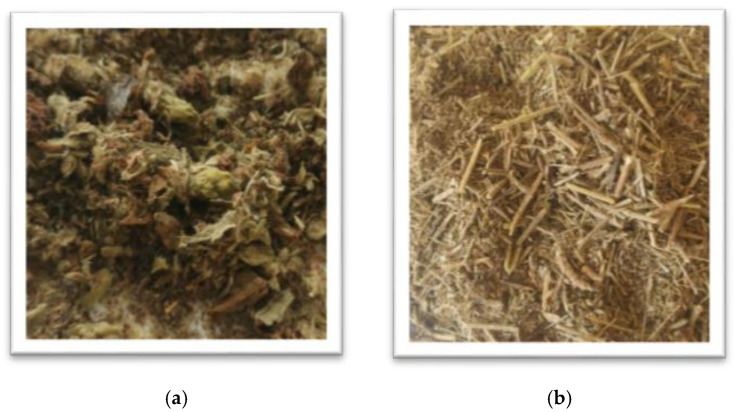
Hexane extracted rose flowers (**a**) and steam distilled lavender straw (**b**).

The hexane extracted rose flowers (*Rosa damascena* Mill.) and the steam distilled lavender straw (*Lavandula angustifolia* Mill.) were provided by Galen*-*N (2020 crop, Zelenikovo distillery, Brezovo region, Bulgaria). The HERF was stored at −18 °C and prior use was dried at 40 °C (Figure 1a) The SDLS was collected immediately after the steam distillation processes from the discarding area near the distillery, air*-*dried at 40 °C and stored at room temperature (Figure 1b). Before use, the SDLS was milled and sieved (particle size 1–5 mm).

#### 2.1.2. Fungal Isolate

The fruiting body of the newly isolated basidiomycete was collected from a forest near Maritza River, Bulgaria in May 2019. In order to isolate a pure culture, the fruiting body was first rinsed very well with tap water and cut into 20 to 30 mm pieces. They were then surface sterilized with 70% ethanol for 20 sec, followed by 10 min in 0.1% Ca(ClO)_2_ and finally rinsed in sterile distilled water. The samples were further sliced to 2.5 by 2.5 mm pieces with sterile scalpel and aseptically transferred on Rose Bengal Chloramphenicol Agar (RBCA) (HIMEDIA, India). The plates were incubated in darkness at 28 °C for 14 days and were visually monitored daily. The fungal culture was isolated and purified by several transfers of growing mycelium on fresh medium. The pure fungal culture was maintained at 4 °C on Mushroom Complete medium (MCM), containing g/L: glucose—20, KH_2_PO_4_—0.5, K_2_HPO_4_—1.0, MgSO_4_—0.5, peptone—2.0, yeast extract—2.0, Agar—2.0, pH 4.8–5.2.

### 2.2. Substrate Preparation Procedure

#### 2.2.1. Preparation for Cultivation

150 g of feeding substrate (SDLS or HERF) was moisturized to final humidity of 65% with a solution containing (g/L): MgSO_4_—0.5, KH_2_PO_4_—0.5, K_2_HPO_4_—1.0, peptone—2.0, yeast extract—2.0. After the addition of 0.1% CaCO_3_ the substrate was mixed well and transferred to autoclavable mushroom growing bags (SacO_2_, Belgium) (100 × 350 mm) and sterilized at 121 °C for 45 min to render the substrate inert. The substrate was left to cool down for 12 h and was used as feeding substrates for solid*-*state cultivation.

#### 2.2.2. Preparation for Chemical Analysis

SDLS or HERF (30 g), milled and sieved (fraction with average particle size 1.5–1.6 mm was used), was treated with preheated 150 mL 70% ethanol for 1 h at 60 °C with constant stirring (120 rpm), then left for 24 h at room temperature (20 °C). The biomass was filtered through cloth (250 mesh), and the remaining residue was treated with additional 100 mL 70% ethanol at the same conditions. After the second filtration, the extracts were combined and used for analyses.

### 2.3. Chemical Characterization of the Substrate

#### 2.3.1. Proximate Composition Determination

The total dietary fibers were determined using K*-*TDFR–100A (Megazyme, Wicklow, Ireland), according to AOAC method 991.43 “Total, soluble and insoluble dietary fibers in foods” (First action 1991) and AACC method 32–07.01 “Determination of soluble, insoluble and total dietary fibers in foods and food products” (Final approval 10–16–91). The uronic acid, cellulose, lignin and non*-*cellulosic polysaccharides in the SDLS and HERF were determined according to Pla et al. [31]. Inorganic matters (ash) were determined after ashing of a 5 g sample at 605 °C.

#### 2.3.2. Determination of Total and Individual Polyphenols

The total polyphenol content was determined according to Singleton and Rossi [32] using Folin*-*Ciocalteu’s reagent (Sigma*-*Aldrich, Schaffhausen, Switzerland). Gallic acid (Sigma*-*Aldrich, Steinheim, Germany) was employed for calibration and the results were expressed as gallic acid equivalents (GAE) per gram dry matter of the extracts. HPLC analyses of the phenolic components were performed on an Agilent 1220 HPLC system (Agilent Technology, Santa Clara, CA, USA), equipped with a binary pump and UV*-*Vis detector. A wavelength of 280 nm was used. The separation of phenolic compounds was performed using an Agilent TC–C18 column (5 µm, 4.6 mm × 250 mm) at 25 °C. The mobile phases consisted of 0.5% acetic acid (A) and 100% acetonitrile (B) at a flow rate of 0.8 mL/min. The gradient conditions started with 14% B, increased linearly to 25% B between the 6th and the 30th min, then to 50% B at the 40th min. The standard compounds (gallic acid, 3,4*-*dihydroxy benzoic acid, chlorogenic acid, caffeic acid, p*-*coumaric acid, ferulic acid, ellagic acid, catechin, epicatechin, rutin, naringin, myricetin, quercetin, naringenin and kaempferol) were purchased from Sigma*-*Aldrich (Steinheim, Germany).

The individual volatile and non*-*volatile compounds in the rose and lavender ethanol extracts were determined according to the following procedures:

(1) Non*-*volatile substances—0.2 mL ethanol extract was lyophilized and 50 μL pyridine and 50 μL N,O*-*Bis*-*(trimethylsilyl)*-*trifluoroacetamide (BSTFA) were added. The sample was incubated at 70 °C for 40 min. For analysis 1.0 μL from the solution was injected on a gas chromatograph Agilent GC 7890 with mas*-*selective detector Agilent MD 5975 and column HP*-*5ms (30 m with diameter 0.32 mm and 0.25 μm thicknesses). The following temperature regimen was used—initial temperature 100 °C (hold for 2 min) then increase to 180 °C with 15 °C/min (hold for 1 min) and increase of the temperature to 300 °C with 5 °C/min (hold for 10 min); injector and detector temperatures—250 °C, helium was used s carrier gas at 1.0 mL/min. The scanning range of the mass*-*selective detector was m/z = 50–550 in split*-*split mode (10:1).
(2) Volatile substances—1.0 mL ethanol extract was extracted with 1.0 mL dichloromethane (triple). The combined organic layers were dried under vacuum at 30 °C. To the dry residue 100 μL dichloromethane was added. For analysis 1.0 μL from the solution was injected on a gas chromatograph Agilent GC 7890 with mas*-*selective detector Agilent MD 5975 and column HP*-*5ms. The following temperature regimen was used—the initial temperature was 40 °C and then increase to 300 °C with 5 °C/min (hold for 10 min); injector and detector temperatures—250 °C, helium was used as carrier gas at 1.0 mL/min. The scanning range of the mass*-*selective detector was m/z = 40–400 in splitless mode. The individual compounds were identified by comparing the retention times and the relative index (RI) with those of standard substances and mas*-*spectral data from libraries of The Golm Metabolome Database (http://csbdb.mpimp-golm.mpg.de/csbdb/gmd/gmd.html, accessed on 5 November 2020) and NIST’08 (National Institute of Standards and Technology, Gaithersburg, MD, USA).

### 2.4. Molecular Identification of the Basidiomycete Isolate by ITS1-5.8S-ITS2 rRNA Gene Sequence Analysis

Prior to DNA extraction, the basidiomycete isolate was cultivated for 7 days on MCM agar plates. The fungal mycelium was scraped out with a sterile spatula (100–300 mg) and transferred to a 2 mL microtube. The DNA extraction was conducted by a modified CTAB method, according to Stefanova et al. [33]. The quality and concentration of the DNA extracts were determined by spectrophotometric measurements using Shimadzu UV*-*VIS spectrophotometer (Shimadzu Corporation, Japan). The ITS*-*5.8S*-*ITS2 region was amplified by forward primer ITS 4 (5′*-*TCCTCCGCTTATTGATATGC*-*3′) and reverse primer ITS 5 (5′*-*GGAAGTAAAAGTGCTAACAAGG*-*3′) (Metabion, Martinsried, Germany) [34]. The PCR reaction mix contained 1 μL of DNA (50 ng), 0.5 μM of each primer and 8 μL of Red*-*Taq DNA Polymerase Master Mix (Canvax Biotech, S.L., Córdoba, Spain) in total volume of 20 μL. The amplification was carried out in a PCR 2720 Thermal Cycler (Applied Biosystems, Waltham, MA, USA) using the following program: initial denaturation at 95 °C for 10 min, followed by 35 cycles of denaturing at 94 °C for 1 min, annealing at 52 °C for 1 min, extension at 72 °C for 1 min, and final extension at 72 °C for 7 min. The PCR product was visualized in 1% agarose gel stained with SafeView (NBS Biologicals, Huntingdon, UK) at 100 V for 50 min using VWR Mini Electrophoresis System (VWR, Darmstadt, Germany) and MiniBis Pro (DNR Bio*-*Imaging Systems, Israel) for gel visualization. The PCR product was cut out from the gel and purified with Clean*-*Easy™ Agarose Purification Kit (Canvax Biotech, S.L., Córdoba, Spain). The sequencing of the PCR product was performed by Microsynth Seqlab (Göttingen, Germany). The resulting sequence was analyzed using BLAST algorithm [35] and compared to the nucleotide sequences in the GenBank database (www.ncbi.nlm.nih.gov, accessed on 21 April 2021). The ITS1*-*5.8S*-*ITS2 rRNA gene sequence of *Ganoderma resinaceum* GA1M was deposited in the GenBank database and an accession number was assigned.

### 2.5. Solid-State Cultivation for Mycelium Bio-Composite Obtaining

#### 2.5.1. Inoculum Preparation

The vegetative inoculum was prepared to form a 7*-*day culture of *G. resinaceum* grown on MCM slant*-*agar. Each 500 mL Erlenmeyer flask, containing 100 mL MCM broth, was inoculated with vegetative biomass from a single MCM*-*slant culture and incubated at 28 °C and 220 rpm for 7 days for pellets formation. The pellets were harvested by sterile filtration and were used for inoculation of the SDLS and HERF feeding substrates. The dry weight of the pellets was determined on moisture analyzer MAC 50/NH (RADWAG, Radom, Poland).

#### 2.5.2. Inoculation of the Substrates and Mycelium Growth

The pellets (10% *w*/*w*) were mixed with the substrates aseptically and left to grow in the mushroom growing bags in darkness at 25 °C for 7 days until the mycelium completely enveloped the substrate’s particles. Every day the mixtures were visually monitored and mixed further to ensure visible uniform mycelium growth and full coverage of the particles of the substrates and the formation of “pre*-*grown” substrate.

**Figure 2:**
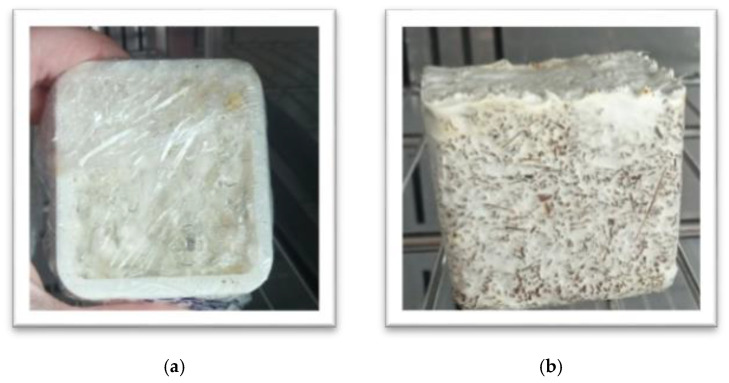
Cultivation of *G. resinaceum* for obtaining mycelium*-*based bio*-*composites: in moulds (**a**) and de*-*moulded (**b**).

The desired sample geometry was achieved as the ready “pre*-*grown” substrates were aseptically transferred into 3D*-*printed molds (55 × 55 × 55 mm), made of poly*-*lactic acid (PLA), consisting of two parts to ensure easy removal of the ready composites. They were manually filled tightly by layering to produce compact and dense samples and then covered with transparent perforated foil to create a microenvironment with high humidity (about 98%), necessary for fungal respiration rate, and incubated at 25 °C, 95% humidity in Nüve climatic chamber (Nüve, Turkey) (Figure 2a). The samples reached the required shape and size after 7 days. Then they were de*-*molded (Figure 2b) and the material was put back in the incubation chamber to grow for another 6 days in order to develop full external homogeneous growth on the sides and to form an external mycelium skin.

The samples were dried in a drying oven SLW 32 (POL*-*EKO*-*APARATURA, Poland) at 60 °C for 8 h in order to remove the moisture and inactivate the mycelium. After drying out, the samples of the two types of mycelium bio*-*composites (HERF*-*based and SDLS*-*based) had the approximate size of 40 × 40 × 40 mm and were used for further tests.

### 2.6. Scanning Electron Microscopy (SEM)

The visualization of the surface and core morphology of the dried mycelium*-*based bio*-*composites was performed by digitized scanning electron microscope (SEM) Philips 515 (Philips, Netherland) in secondary electrons imaging (SEI) mode. All analyses were performed at an accelerating voltage of 8 kV and different magnifications between 200× and 5000×. A preliminary preparation of the samples was applied, including fixation of pieces of the corresponding composites samples on standard SEM stubs and subsequent metallization of their surfaces by deposition of an Au film.

### 2.7. Fourier Transform Infrared Spectroscopy (FTIR)

The FTIR spectra of the feeding substrates SDLS and HERF and the obtained SDLS and HERF*-*based mycelium bio*-*composites previously ground and homogenized (2 mg) were collected on a Fourier transform infrared (FTIR) spectrophotometer VERTEX 70v (Brucker, Germany) in KBr pellets (Honeywell/Fluka FTIR grade > 99%). The spectra were recorded in the 4000–400 cm^−1^ range at 132 scans with a spectral resolution of 2 cm^−1^. Three samples of each type were measured to ensure reproducibility.

### 2.8. Basic Physical and Mechanical Characterization

The apparent density was determined according to ISO 29470:2020. The short and long*-*term water absorption was measured according to ISO 29767:2019 and ISO 16535:2019, respectively. EN 826:2013 was used for compression behavior determination. Three samples of approximate size 40 × 40 × 40 mm were used for each test of the mycelium HERF*-* and SDLS*-*based bio*-*composites. The average value and the standard deviation were calculated. Despite the structural differences between the core and the near*-*surface zones of the samples, the testing was performed on intact samples, in order to take into consideration the specific properties of bio*-*composites due to the mycelium “coating” of the samples. Thus, the results reflected the overall behavior, but were highly influenced by the surface/volume ratio. In case the bio*-*composites are to be used in applications where the edges need to be trimmed or the surface layer removed, the tests must be carried out on the relevant representative samples.

The parallel piped samples were dried out in a ventilated oven at +60 °C until constant mass md (precision of 0.01 g) was reached. The dimensions were measured by a caliper with a precision of 0.01 mm. Each dimension (length, width, height) was measured three times—at the edges and in the middle and the average value was reported. The apparent density of each sample was calculated by the formula:

(1) $$\rho_{a}=\frac{m_{d}\;}{a.b.h}$$

where, *ρ_a_* is the apparent dry density, in kg/m^3^; *m_d_* is the mass in dry condition, in kg and *a*, *b*, *h* are the dimensions (length, width and height) of the samples in dry condition, in m.

The capillary absorption was measured by a partial immersion (1 cm of the height of samples) in water at +20 ± 3 °C. The samples were placed on a grid, allowing free water circulation below the sample surface. The water level was maintained constant for 24 h. After removal from the water the surface was wiped off with a moist cloth and the mass of each sample was then measured. The calculations were made according to Formula (2):

(2) $$W_{c}=\frac{m_{w,c}-m_{d}\;}{a.b}$$

where *W_c_* is the capillary absorption per unit area for 24 h, in kg/m^2^; *m_d_* is the mass in dry condition, in kg; *m_w,c_* is the mass in wet condition after 24 h of partial immersion in water, in kg and *a, b* are the width and length of the samples, in m.

Water absorption was measured after short*-*term (1 day) and long*-*term (28 days) immersion in water. The samples were placed between 2 grids and covered with 1 cm of water at +20 ± 3 °C. The bottom grid allowed free circulation of water, while the upper grid served as a support for additional weights on the samples in order to avoid their emergence. After removal from the water the surface was wiped off with a moist cloth and the mass of each sample was then measured.

The water absorption was calculated as follows:

(3) $$W^{m}{}_{a,1d}=\frac{m_{w,1}-m_{d}\;}{m_{d}}.100$$

(4) $$W^{m}{}_{a,28d}=\frac{m_{w,28}-m_{d}\;}{m_{d}}.100$$

(5) $$W^{v}{}_{a,1d}=\frac{\left(m_{w,1}-m_{d}\right)/\rho_{w}\;}{a.b.h}.100$$

(6) $$W^{v}{}_{a,28d}=\frac{(m_{w,28}-m_{d})/\rho_{w}\;}{a.b.h}.100$$

where *W^m^_a,1d_* is the short term water absorption after 1 day of full immersion in water, expressed per unit mass, in % wt; *W^v^_a,1d_* is the short term water absorption after 1 day of full immersion in water, expressed per unit volume, in % vol.; *W^m^_a,28d_* is the long*-*term water absorption after 28 days of full immersion in water, expressed per unit mass, in % wt.; *W^v^_a,28d_* is the long term water absorption after 28 days of full immersion in water, expressed per unit volume, in % vol.; *m_d_* is the mass in dry condition, in kg; *m_w,1d_* is the mass in wet condition after 1 day of full immersion in water, in kg; *m_w,28d_* is the mass in wet condition after 28 days of full immersion in water, in kg; *a*, *b*, *h* are the dimensions (length, width and height) of the samples in dry condition, in m and *ρ_w_* is the density of water at +20 °C, i.e., 997 kg/m^3^.

The compressive stress at 10% relative deformation was calculated as follows:

(7) $$\sigma_{10}=\frac{F_{10}}{a.b}$$

where, *σ_10_* is the compressive stress at 10% deformation, in MPa; *F_10_* is the load at which the test specimen reaches the required longitudinal deformation of 10%, in N and *a*, *b* are the width and length of the samples, in mm.

### 2.9. Statistical Analysis

All the experiments were conducted in triplicate and the values were expressed as mean ± SD. Statistical significance was detected by analysis of variance (ANOVA, Tukey’s test; value of *p* < 0.05 indicated statistical difference).

## 3. Results and Discussion

### 3.1. Chemical Characterization of the HERF and SDLS and Assessment of Their Potential as Feedstock for Mycelium Growth and New Bio-Materials Development

The selection of HERF and SDLS as substrates in this study was mainly based on the fact that they are generated globally and in Bulgaria in significant amounts as essential oil industry waste by*-*products. The solid rose and lavender processed biomass are underutilized and mostly thrown away by distilleries. These circumstances are a prerequisite for the sustainable valorization of rose and lavender by*-*products and their transformation into new mycelium bio*-*composites with added value. Moreover, the industrial processing of essential oil plants (steam and steam*-*water distillation, extraction with organic solvents or supercritical gases) leads naturally to sterilization, which could simplify the procedure for mushroom growth substrate preparation.

Knowing the chemical composition of these waste materials is of critical importance not only to the stimulation of the vegetative growth and development of the basidiomycete strain but to the formation of a homogenous mycelium bio*-*composite with proper integrity and innovative aromatic and bactericidal properties.

The proximate composition of the HERF and SDLS revealed that there were significant differences in the analyzed compound content (Table 1). The concentration of lignocellulose carbohydrates in both studied substrates was higher than that of non*-*cellulosic polysaccharides. Higher content of cellulose and lignin was determined in SDLS—38.16 and 24.48 g/100 g DW, respectively. In HERF the concentrations of cellulose and lignin were about 24% lower and were 29.13 and 18.57 g/100 g DW, respectively. This is dependent on the type of raw material (rose and lavender) used by the distilleries. Lavender is a crop that is much easier to harvest mechanically and the whole stem is mowed, while only the valuable part of the roses—the flower, is harvested. This explains the higher content of cellulose and lignin in SDLS. It is well known that the inner part of the rose flower is a good source of pectic polysaccharides [36], which was reflected in the higher uronic acid concentration in the HERF (8.95 g/100 g DW) in comparison with the 3.54 g/100 g DW for SDLS. The difference in the concentration of non*-*cellulosic polysaccharides was small—14.57 g/100 g DW in HERF and 13.79 g/100 g DW in SDLS. The lignin content of SDLS (24.48%) and HERF (18.57%) was closer to that of softwood (18–26%) and grass (17–24%) than the one of hardwoods (27–33%) [37].

The variation of the type and initial concentration of polysaccharides in both studied substrates is an important factor affecting the mycelium fungal growth, the colonization rate of the substrates and the physico*-*mechanical properties of the obtained mycelium*-*based composite.

The content of lignocellulose compounds, together with the non*-*cellulosic polysaccharides and uronic acid could act as an essential carbon source for the vegetative development of the studied basidiomycete strain. Non*-*cellulosic polysaccharides and uronic acid would probably stimulate the initial fungal biomass production and later partial de*-*polymerization of lignin and cellulose would occur. According to Singh and Singh [38], basidiomycetes have their own mechanism for penetration of lignocellulose materials starting through nutrient*-*rich parenchyma cells, which are composed of easily metabolized components. Further, due to the thin hyphae formation and synthesis of a specific ligninolytic enzyme system, they can bore through the cell wall and support full colonization of the lignocellulose substrates.

The lignin and cellulose content and lignocellulose fibers reinforce mycelium*-*based bio*-*composite materials because of the crystallinity of cellulose and the protecting role of lignin in the lignocellulose matrix [39]. A large number of various chemical groups in the lignin macromolecules is a prerequisite for modifications and new functional groups occurrence by enzymatic or biological methods to improve adhesion and to support aggregation of lignocellulose particles [40]. The presence of lignin also helps to improve the stiffness of the bio*-*composites and protecting the ready bio*-*materials against microbiological attack [39].

The potential substrates were further analyzed for the presence and quantity of polyphenol compounds (total and individual) and the results are presented in Table 2.

Both rose and lavender essential oil industry by*-*products were found to be rich in polyphenols [3,41]. The fivefold difference in the total polyphenol content: 5.70 and 1.14 g/100 g DW for HERF and SDLS, respectively, is mainly due to the differences in industrial processing. The fresh rose flowers were extracted with a non*-*polar solvent (hexane), which resulted in the preservation of a large part of the polyphenols in the residual biomass, while the lavender was steam*-*distilled leading to partial extraction of phenol compounds. The highest amounts of individual polyphenols in HERF were found for rutin and quercetin*-*3*-*β*-*glucoside (glycosides, which are poorly soluble in hexane), while in SDLS 3,4*-*dihydroxy benzoic acid and gallic acids (0.51 and 0.29 g/100 g DW, respectively) were predominately identified.

The presence of polyphenols in the rose and lavender by*-*products could influence positively the mycelium growth because they might be used as a carbon source on account of the enzyme*-*oxidizing system of the basidiomycete isolate. The polyphenol content also suggests bacteriostatic and bactericidal effects of the substrates, which could support the initial sterilization procedure, as well as serve as a natural bio*-*protective barrier against bacterial contamination in the process of bio*-*composite production and application.

The process of essential oil production (either by extraction, or distillation) is never exhausting and part of the aroma compounds remain in the solid or liquid by*-*products. These aroma substances, mainly terpenes, often possess antimicrobial properties and could also serve as bio*-*preservative agents. Their presence in the plant matrix could contribute to the final pleasant aroma of the mycelium*-*based bio*-*composites. Phenethyl alcohol, β*-*citronellol, geraniol, β*-*cubebene, β*-*linalool and nerol were found in higher amounts in the HERF and β*-*linalool, dihydro*-*linalyl acetate, lavandulol, α and β*-*caryophyllene, lavandulyl acetate and α*-*terpineol were distinctive for the SDLS by*-*products (Table 3).

The chemical characteristics of SDLS and HERF showed potential to stimulate mycelium growth of the basidiomycete isolate and to support self*-*growth of mycelium bio*-*materials with improved aroma and bactericidal properties.

The use of HERF and SDLS as a feedstock for new mycelium*-*based bio*-*composite production could be accepted as an innovation because at present there are no reports on the usage of these by*-*products for mycelium*-*based bio*-*composite formation. The chemical composition of the SDLS and HERF is a serious prerequisite for the development of mycelium*-*based bio*-*composites with improved antibacterial and aromatic properties.

### 3.2. Solid State Cultivation of Ganoderma Resinaceum GA1M on HERF and SDLS for Mycelium-Based Bio-Composite Formation

The molecular identification of the new regionally isolated basidiomycete was performed by amplification of the ITS1*-*5.8S*-*ITS2 region and the obtained PCR product was subjected to sequence analysis. The resulting sequence was analyzed using BLAST algorithm and compared to the nucleotide sequences in the GenBank database (www.ncbi.nlm.nih.gov, accessed on 10 September 2021). The strain was identified with high percent confidence (99.67%) as *Ganoderma resinaceum*. The ITS1*-*5.8S*-*ITS2 rRNA gene sequence *of G. resinaceum* GA1M was deposited in the GenBank under accession number MW996753.

*G. resinaceum* belongs to the white*-*rot fungi (*Basidiomycota, Polyporales*) and is most commonly found as a saprophyte on wood trunks, but also as a parasite on dying trees, mainly in forests in the temperate zone. In Bulgaria, this fungus grows most often on deciduous trees and in rare cases on conifers.

Our interest in *G. resinaceum* for the present experimental research was based on preliminary results from the investigation of the growth rate of this strain’s mycelium on different lignocellulose materials (the data are not published). It exhibited relatively fast growth, which was suitable for the purposes of this study.

**Figure 3:**
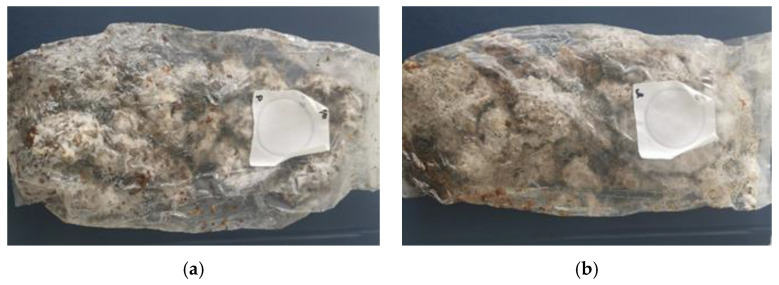
“Pre*-*growth” substrates—hexane extracted rose flowers (**a**) and steam distilled lavender straw (**b**). The cultivation of “pre*-*growth” substrates went on into special molds for another 7 days, where the fungal strain continued to increase its mycelial mass, to synthesize enzymes degrading the substrate polysaccharides and to produce metabolites, which additionally support the binding of the lignocellulose fibers of the substrate, envelope them and contribute to the final shape and structure of the biomaterial (Figure 2a).

In the development of new bio*-*composites, the whole process was based mainly on the solid*-*state cultivation of *G. resinaceum* on HERF and SDLS and comprised of several stages. The strain utilization of the studied substrates started with relatively rapid growth on both substrates resulting in the formation of a dense well*-*developed mycelium network and almost full colonization was observed. Mycelium bio*-*composites with a homogeneous structure and proper integrity were obtained after 20 days. The first sign of fungal growth was detected on the surface of HERF (Figure 3a) 48 h after inoculation while the visual growth on SDLS (Figure 3b) started after 72 h. Although *G. resinaceum* showed a slight delay in growth on lavender straw waste during the first 72 h, compared to rose flowers, fully homogenous “pre*-*growth” substrates were produced after 7 days of cultivation for both substrates.

**Figure 4:**
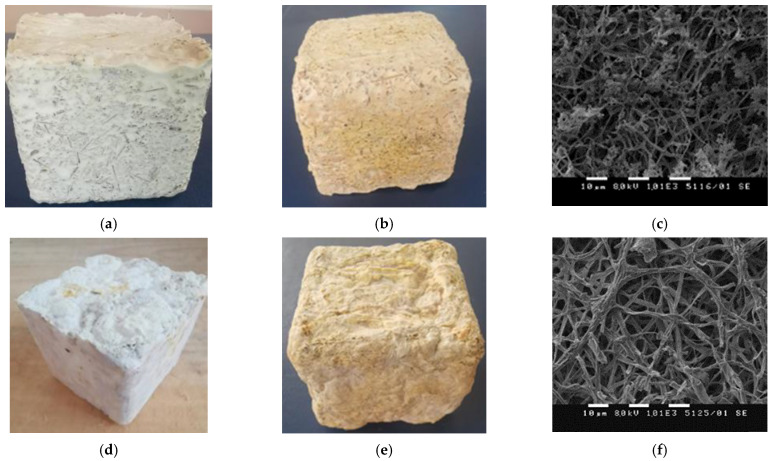
Mycelium steam distilled lavender straw (SDLS)*-*based bio*-*composites grown for 20 days before (**a**) and after drying at 60 °C for 8 h (**b**), Scanning Electron Microscopy (SEM) image of mycelium surface layer (**c**); mycelium hexane extracted rose flowers (HERF)*-*based bio*-*composites grown for 20 days before (**d**) and after drying at 60 °C for 8 h (**e**), SEM image of mycelium surface layer (**f**).

The visual observation showed that the strain grew very well on the top surface of both studied substrates, which were in contact with the air, and formed velvety aerial mycelium, resembling skin. At the bottom and on the sides, which were in contact with the mold, the mycelium was less dense than that at the top surface (Figure 2b). When the cultivation period was completed, the substrate surfaces were coated with a white mycelial layer with small brownish spots, and had a relatively regular three*-*dimensional shape (Figure 4a,d). The brownish spots are commonly reported and accepted as a result of the de*-*polymerization of polysaccharides and the melanin production in the early stages of lignocellulose substrates degradation [4,27]. The moisture content of the substrate and the relative humidity of the air during the vegetative growth stage had a direct effect on the mycelium growth given that they influence both nutrient availability and fungal degradation. When the mycelium reached optimal growth and fully covered and bound the fibers of the substrates, the samples were dried at 60 °C for 8 h to cease fungal growth and remove the moisture. During this process, the mycelium adhered tightly to the fibers of the substrate and as a result, mycelium HERF/SDLS*-*based bio*-*composites with specific visual characteristics were obtained.

As presented in Figure 4b,e, both materials had acquired a yellowish color of the mycelium layer after drying compared to the color before. According to Apples et al. [22], the change in color during the process of drying or thermal pressing is common and is probably due to Maillard reactions between the sugars and proteins present in the fungal cell walls and the plant materials or it is caused by low water content in the area.

Visually both mycelium*-*based materials appeared to have randomly oriented fibers tightly covered by fungal mycelium. The SDLS*-*based bio*-*composite showed bigger conformational stability and less deformation after drying compared to the HERF*-*based one. This could be explained by the type of used substrates and their cellulose and lignin content. These two polysaccharides are mainly responsible for the conformational stability of the mycelium*-*based bio*-*composites [39]. The better dimensional stability of the SDLS*-*based bio*-*composites was probably a result of the use of the whole lavender straw, while only the flower was used form of the roses. The significant rigidity and sturdiness of the lavender straw supported the conformational stability unlike the fragility of rose flowers. The dimensional stability of rose flower*-*based bio*-*composite could be improved by blending it with more rigid substrates. Both composites had surface texture, which could be described as satiny and velvety to the touch, but the HERF*-*based mycelium bio*-*composite surface was slightly wavier and rougher.

The SEM images of the surface of both mycelium bio*-*composites after drying are shown in Figure 4c,f. They depict a dense and adequately developed mycelium layer, forming a fibrous macro-porous structure. As can be seen in the images, the filaments appeared to flatten. The same findings were reported by Haneef et al. [6]. According to the authors, the thermal treatment for 7 h at 60 °C stopped the mycelium growth and the hyphae were not supported by internal hydrostatic pressure.

**Figure 5:**
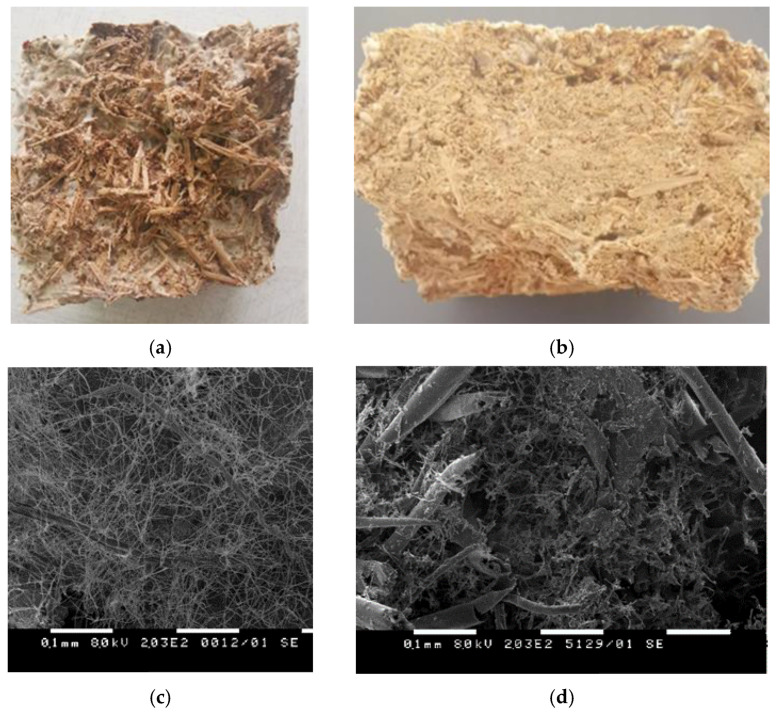
Visual observation of cross*-*section of mycelium (**a**) steam distilled lavender straw (SDLS)*-* and (**b**) hexane extracted rose flowers (HERF)*-*based bio*-*composites; Scanning Electron Microscopy (SEM) images of cross*-*section of (**c**) SDLS*-* and (**d**) HERF*-*based bio*-*composites.

The visual observation of the cross*-*section of both mycelium composites showed that the fungal colonization was denser when closer to the air*-*contacted sides of the materials than in their center (Figure 5). The cross*-*section of the mycelium SDLS*-*based bio*-*composite exhibited more porosity (Figure 5a) and the hyphae had fully colonized the space between the lavender straw fibers at the outer part of the material, but they had not completely penetrated the center. Less clearly visible mycelium growth was observed during the visual inspection of the cross*-*section of the mycelium bio*-*composites made of HERF. The fungal hyphae and fibers looked glued together and the structure of the HERF*-*based bio*-*composite was denser and tougher (Figure 5b). As oxygen stimulates the mycelium growth, the difficulties in air penetration to the center of the material is one of the possible reasons for weak mycelium growth in the core, together with fiber structure and heat production by the mycelium during the degradation processes [9,19,22]. The SEM images confirmed the less porous internal part of the mycelium HERF*-*based bio*-*composite compared to the SDLS*-*based one (Figure 5c,d). According to Lelivelt et al. [42], if the thickness of the material is increased, there will be a point at which the center becomes too hot or too anaerobic to allow any growth at all. As proposed by other authors, a longer cultivation time could improve the colonization of the core of the composite [21,43].

The fungal growth rate, the full coverage of the composite with aerial mycelium and the dense hypha network in the center of the materials influence their physical and mechanical characteristics. They, in turn, determine the application of the developed bio-materials.

### 3.3. FTIR Spectroscopy

**Figure 6:**
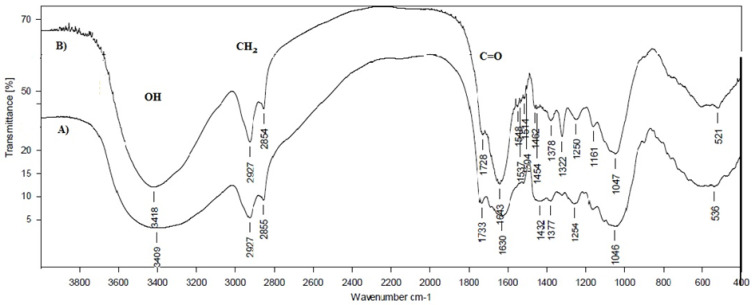
FTIR spectra of steam distilled lavender straw (SDLS) substrate (**A**) and mycelium SDLS*-*based bio*-*composite (**B**).

**Figure 7:**
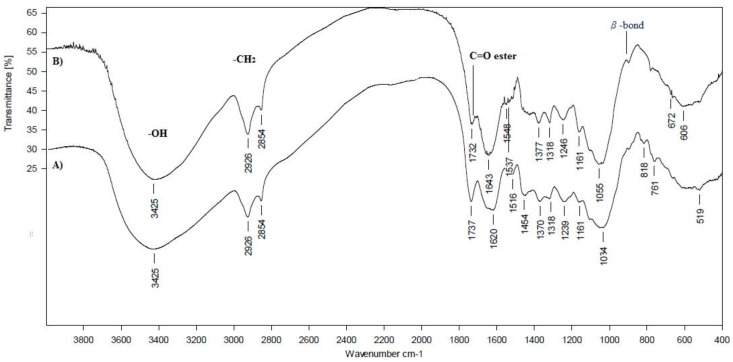
FTIR spectra of hexane extracted rose flowers (HERF) substrate (**A**) and mycelium HERF*-*based bio*-*composite (**B**).

FTIR spectroscopy was used to characterize the functional groups of both substrates (HERF and SDLS) and the obtained mycelium*-*based bio*-*composites. The FTIR spectra of the mycelium HERF/SDLS*-*based bio*-*composites were compared to the initial HERF and SDLS substrates (Figure 6 and Figure 7). FTIR bands were assigned in detail taking note of literature data for other mycelium*-*based bio*-*composites produced on the base of different combinations between substrates and basidiomycetes (Table 4). In general, the infrared absorption spectra of the mycelium bio*-*composites were associated with the biomolecules that compose them, e.g., lipids (3000–2800 cm^−1^, ~1737 cm^−1^ ester bonds), proteins (amide I at 1700–1600 cm^−1^, amide II and III at 1575–1300 cm^−1^), nucleic acids (1255–1245 cm^−1^), and polysaccharides (1200–900 cm^−1^), which was in accordance with the bands previously reported in the literature [6,17,40].

The ratio of the band intensity of the absorption associated with the C*-*H bending mode of chitin (~1374 cm^−1^) to the one of the C*-*C stretching of polysaccharides (~1043 cm^−1^) remained constant and unchanged. The IR bands in the region of 1000–1200 cm^−1^ were related to stretching vibrations of C*-*O*-*C and C*-*O, respectively. The FTIR bands at 847 cm^−1^ characterized the β*-*1,4 bonds vibrations. In our case, the presence of β*-*bonds in the mycelium HERF*-* and SDLS*-*based bio*-*composites was clearly observed (Table 4 and Figure 6 and Figure 7). Bruscato et al. [17] also reported that the bands between 915 and 1110 cm^−1^ were assigned to the D*-*Glc*p* unit and β*-*configuration of the sugar units in the polysaccharide at 890 cm^−1^. In our study, the β*-*configuration of the sugar units in the polysaccharide matrix was preserved and these bonds did not disappear (Table 4).

The degradation of lavender straw and rose flower waste by *G. resinaceum* led to a small decrease in the intensities of carbohydrates at 1733 cm^−1^, 1158 cm^−1^, 897 cm^−1^ and a similar observation was reported for the flax composites produced by *T. versicolor* [40].

Relative increases in protein and lipid bands were detected in both mycelium HERF*-* and SDLS*-*based bio*-*composites, compared to the initial substrates. A large band (18 cm^−1^, from 1686 to 1668 cm^−1^ to lower wavenumbers) was ascribed to the amide I of β*-*turns [6]. In addition, the bands at 1548 cm^−1^, typical for amide II, were found only in mycelium grown on HERF and SDLS and were completely absent in the used waste substrates (Table 4). Bands due to the interaction of the polysaccharides with the mycelium at 1551 cm^−1^ (lignin bands) and 1318 cm^−1^ (cellulose bands) were not found in the mycelium HERF/SDLS*-*based bio*-*composites (Table 4). Contrary to the reports of Elsacker et al. [40], in our study, the decrease in carbohydrates (especially cellulose) was most pronounced at 1317 cm^−1^ (strong vibration) as a result of *G. resinaceum* growth. However, the FTIR spectra also revealed a small band at 1377*-*1378 cm^−1^ (weak vibrations), assigned to chitin. The ratio of bands intensities between the mycelium HERF/SDLS*-*based bio*-*composites and the HERF and SDLS substrates (1510 cm^−1^/1510 cm^−1^) was 3,6 for SDLS and 1.67 for HERF. This ratio revealed a higher intensity of lignin band for the SDLS*-*based bio*-*composite than the lavender straw. In comparison, Elsacker et al. [40] reported, that the depolymerisation of lignin by *T. versicolor* occurred to a greater extent in flax (0.97) than in hemp (0.57).

Haneef et al. [6] compared the FTIR spectra of the mycelium species, independent of the feeding substrates, and found that *G. lucidum* showed a higher contribution of lipids, whereas *Pleurotus ostreatus* showed relatively more intense bands that were due to polysaccharides. Similar to FTIR bands obtained from *Pycnoporus sanguineus*, *P. albidus* and *Lentinus velutinus* growth on wheat bran [17], the bands of the bio*-*composites from SDLS and HERF could not be observed, probably because they were consumed due to overlap with bands of cellulose, hemicellulose or lignin.

Comparing the data from the current research with reference literature data, it is clear that the chemical nature of the substrates is also responsible for distinct changes in the FTIR spectra of the mycelium*-*based bio*-*material. In conclusion, a decrease in cellulose bands and decomposition of lignin were observed and amide II bands appeared, which confirmed the use of HERF and SDLS as substrates for the *G. resinaceum* growth in both mycelium bio*-*composites.

### 3.4. Basic Physical and Mechanical Characterization of the Mycelium HERF/SDLS-Based Bio-Composites

Taking into consideration the physical structure and possible application of the mycelium*-*based bio*-*composites, standardized methods for thermal insulating products for building applications were used for the determination of some basic physical and mechanical properties. The results are summarized in Table 5. The apparent density of the materials is considered as one of the main parameters, allowing the prediction of other significant characteristics (porosity, water absorption, strength, thermal conductivity, etc.) and it is a good indicator of the mechanical properties [11,20,44]. For mycelium*-*based bio*-*composites, it has also been established that density is an important factor regarding the competitiveness of these materials and their applications [23]. Our analyses revealed that the mycelium HERF*-*based bio*-*composite had higher density (462 kg/m^3^) as compared to the SDLS*-*based bio*-*composite (347 kg/m^3^). These results reflect the porosity of the mycelium bio*-*composites, the differences in the chemical composition and particles sizes and distribution of the used waste rose flowers and lavender straw together with the mycelium growth.

Based on literature data about the density of natural lignocellulose substrates varying from 1.2 to 1.5 g/cm^3^ [44], porosity is considered to be a significant factor that could affect the densities of mycelium*-*based composites [23]. The cross*-*section of the SDLS*-*based mycelium composites (Figure 5a) showed the presence of more macro*-*pores allowing a bigger amount of hyphae inside the material. The structure of the HERF*-*based bio*-*composites was more compact and tougher, especially in the core of samples, thus leading to a higher apparent density of the mycelium HERF*-*based bio*-*composites. (Figure 5b). Our findings of the higher apparent density of the mycelium HERF*-*based bio*-*composites in comparison with the SDLS*-*based bio*-*composite is in contrast with a previous study [6] according to which higher amount of branched hyphae led to a more compact structure and higher density of the developed mycelium*-*based bio*-*composites. Our findings are in conformity with those of Tacer*-*Caba et al. [20], according to which fewer branched hyphae of *P. ostreatus* grown on oat husk and rapeseed cake resulted in a denser material. The degradation of lignocellulose substrates and their substitution with fungal biomass caused a decrease in the bio*-*composite density because the reported density of pure mycelium is quite low 0.03–0.05 g/cm^3^ [13].

The density of the mycelium HERF/SDLS*-*based bio*-*composites was similar to non*-*pressed mycelium*-*based bio*-*composites obtained in different studies using various lignocellulose substrates, which vary from 59 to 590 kg/m^3^ [7,8,9,17,22]. The mycelium HERF/SDLS*-*based bio*-*composites were denser than expanded polystyrene (EPS) (22–50 kg/m^3^) [20,23] and lighter in comparison with wood*-*based composites such as medium*-*density fiber board (MDF) (500–1000 kg/m^3^) and oriented strand board (OSB) (550–700 kg/m^3^) [22]. The density of the HERF/SDLS*-*based mycelium bio*-*composites was also similar to hempcrete, which is between 400–500 kg/m^3^ and 200–250 kg/m^3^, obtained by on*-*site pouring and spray method, respectively [45].

Depending on the intended use of the mycelium HERF/SDLS bio*-*composites—for non*-*structural or semi*-*structural applications (i.e., thermal insulation or particleboards), the density might be further decreased by mixing the HERF and SDLS with other lignocellulose substrates with low density and an increase in the fungal biomass in the core of the bio*-*composites or it could be increased by cold or thermal pressing.

Water absorption is another very important parameter that influences quality, durability and the application range of a mycelium*-*based bio*-*composite [40]. The capillary absorption, short*-*term water absorption and long*-*term water absorption of the HERF/SDLS*-*based mycelium bio*-*composites were evaluated.

Water absorption is the easiest way to characterize the open porosity of materials, especially the absorption through capillarity. As can be seen in Table 5, the capillary absorption of the HERF*-*based mycelium bio*-*composites was about two times lower (3.4 kg/m^2^) than the SDLS*-*based mycelium bio*-*composite (6.5 kg/m^2^), which confirmed that the HERF*-*based mycelium bio*-*composites had a smaller amount of coarse pores than the SDLS*-*based ones. The obtained results were higher than those reported by Elsacker et al. [40] for a mycelium chopped hemp, flax*-* and straw*-*based bio*-*composite varying between 2 and 3.8 kg/m^2^. The results on water absorption after one day of full immersion, which can be taken as representative for the finer porosity, confirmed the same—the water absorption of the mycelium HERF*-*based bio*-*composites was two times lower (ca. 20% vol.), as compared to that of the SDLS*-*based bio*-*composites (ca. 40% vol.). The long*-*term water absorption of the studied bio*-*composites reached 58%vol. (126% wt.) for the HERF*-*based bio*-*composite and 85%vol. (245% wt.) for the SDLS*-*based bio*-*composites. This correlates with reference literature data for bio*-*composites, where values up to 350% wt. were presented [4,24,46]. Apples et al. [22] also reported that the water absorption of different mycelium lignocellulose*-*based bio*-*composites ranged from 43% wt. to 508% wt. after only 8 days of immersion in water. The water absorption of the studied bio*-*composites exceeded considerably the water absorption of some hemp hurds composites, reported to be between 6.3% wt. and 25.8% wt. [47] and was much higher than the long*-*term water absorption of conventional thermal insulating material—between 0.2% vol. for extruded polystyrene (XPS) to 2.8% vol. for EPS, after 2 months of immersion [48]. Thus, it seems the present mycelium bio*-*composites would not be suitable for applications involving frost attack, because in that case the long*-*term water absorption shall be limited to 2%. However, the water absorption of the bio*-*composites could be significantly modified, because it is due not only to the open (to water) porosity, but also to the water suction by the used HERF and SDLS. Further testing shall clarify the impact of water absorption on the bio*-*composites swelling and the mycelium changes. The behavior of mycelium*-*based bio*-*composites during water immersion strongly depends on the hydrophobic properties of the fungal mycelium [20,23] and the hydrophilicity of lignocellulose fibers [46]. The hydrophobicity of basidiomycetes is mainly a result of the content of a low*-*weight protein, called hydrophobin. This protein was also reported to affect the process of adhesion of lignocellulose particles [6,20,23]. To limit the hydrophilicity of the lignocellulose fibers and reduce the water absorbance of the mycelium HERF/SDLS*-*based bio*-*composites, further investigations are needed in order to obtain well*-*developed mycelium, fully enveloped substrates particles together with an intact outer mycelium layer that covers the entire bio*-*composite. Another option would be to cover the bio composites with different bio*-*coatings (chitosan, carrageenan and xanthan), which was reported to considerably decrease the water absorbance [24].

In the preliminary testing of compressive behavior, no fracture was observed, because the bio*-*composites were quite deformable. Therefore, the behavior at compression is characterized by the compressive stress, corresponding to 10% of longitudinal strain, i.e., compressive stress at 10% relative deformation. In order to create smooth and parallel loading surfaces, a preliminary treatment by a thin layer of cement paste was applied and the testing was performed after 48 h. The compressive resistance of the lighter mycelium SDLS*-*based bio*-*composite was 718 kPa, while it was 1029 kPa for the relatively denser mycelium HERF*-*based bio*-*composite. Both values are quite higher than the range of compressive strength of different mycelium*-*based bio*-*composites, reported by Amstivslaski et al. [49], which was between 29 and 567 kPa. It could be concluded that our results also surpassed significantly the typical values for some conventional thermal insulating materials, such as several standard categories of EPS, which do not exceed 120 kPa for short*-*term loading [50]. However, it shall be mentioned that there is a significant impact of the density on the compressive behavior of thermal insulating materials and thus various data can be found. Because of this Nava et al. [24] stated, that the compressive strength of mycelium*-*based bio*-*composites was lower than most of the EPS categories and our results for compressive resistance were closer to the compressive strength of hempcrete, which has a similar apparent density [45].

The experimental data on compressive behavior of the mycelium HERF/SDLS*-*based bio*-*composite are probably due to the successful combination between the used fibrous substrates and the basidiomycete strain *G. resinaceum*. It was observed that the mycelium of *Ganoderma* spp. demonstrated quite robust consistency as compared to other basidiomycetes [24].

Due to the smaller size of the samples than the required by EN 12667:2001, the coefficient of thermal conductivity (lambda) was not measured. A rough estimation on the lambda range was carried out based on the apparent density of the studied bio*-*composites, the available literature data on mycelium*-*based bio*-*composites and data on other similar thermal insulating products.

It was difficult to assess lambda only based on apparent density, because the densities were closer to that of inorganic thermal insulation materials (such as cellular concrete, porous ceramic, etc.), while the bio*-*composites are an organic material, with quite a big volume of open porosity (such as EPS). The thermal insulation with the closest profile is hempcrete, but its binder is inorganic (lime, cement, magnesia binder, etc.). Therefore, the developed mycelial bio*-*composites are quite specific, combining a fibrous component (waste lavender straw and rose flower biomass) and an organic binder (mycelium). Considering only the density values, the maximum thermal conductivity coefficient, based on cellular concrete and hempcrete data, can be expected to be 0.12–0.17 W/m.K for the HERF-based composites and 0.09–0.11 W/m.K for the SDLS bio*-*composites [24]. The study of Jones et al. [11] showed that when the apparent density of mycelium composites varied between 59 and 552 kg/m^3^, the values of lambda varied between 0.04 and 0.18 W/m.K. If thermal insulation application is targeted, the density of the bio-composites should be significantly reduced and lambda might be quite low as well. For density in the range of 95–135 kg/m^3^ the reported data for lambda was between 0.04 and 0.06 W/m.K, i.e., very similar to that of conventional thermal insulating materials [40].

## 4. Conclusions

This is the first study reporting that waste hexane extracted rose flowers and steam distilled lavender straws showed realistic potential to be utilized as a feedstock for new mycelium-based bio-composites development by a new locally isolated and molecularly identified *Ganoderma resinaceum* GA1M. The visual observation and SEM images of the surface and cross-section of the mycelium HERF/SDLS*-*based bio-composites both showed adequately developed aerial mycelium layer, fibrous internal macroporous structure and proper integrity. Basic physical and mechanical characterization of the obtained mycelium HERF/SDLS-based bio-composites revealed promising potential and after additional optimization they could be suggested for non-structural or semi-structural applications and interior design.

## Figures and Tables

**Table 1:** Proximate composition of hexane extracted rose flowers (HERF) and steam distilled lavender straw (SDLS).

	HERF, g/100 g DW	SUM	SDLS, g/100 g DW	SUM
Fibers (total)	75.14 ± 0.19 ^b^	*-*	81.54 ± 0.18 ^a^	*-*
Uronic acids	8.95 ± 0.17 ^a^	*-*	3.54 ± 0.12 ^b^	*-*
Ash	3.10 ± 0.16 ^b^	*-*	6.59 ± 0.11 ^a^	*-*
Non*-*cellulosic polysaccharides	14.57 ± 0.2 ^a^	62.27 ± 0.21 ^b^	13.79 ± 0.15 ^a^	76.43 ± 0.21 ^a^
Cellulose	29.13 ± 0.17 ^b^		38.16 ± 0.21 ^a^	
Lignin	18.57 ± 0.16 ^b^		24.48 ± 0.14 ^a^	

Data were expressed as mean ± SD (*n* = 3); ^a,b^—different letters indicate significantly different values in the rows (Student’s *T* test; value of *p* < 0.05 indicated statistical significance).

**Table 2:** Total polyphenol content, individual flavonoids and phenolic acids in hexane extracted rose flowers (HERF) and steam distilled lavender straw (SDLS).

	HERF, g/100 g DW	SDLS, g/100 g DW
Total polyphenols	5.70 ± 0.20 ^b^	1.14 ± 0.07 ^a^
Quercetin	0.30 ± 0.002 ^b^	0.03 ± 0.001 ^a^
Quercetin*-*3*-*β*-*glucoside	0.48 ± 0.002 ^b^	0.04 ± 0.001 ^a^
Rutin	0.98 ± 0.002	nd
Myricetin	0.09 ± 0.001 ^b^	0.028 ± 0.001 ^a^
Kaempferol	0.04 ± 0.001 ^b^	0.01 ± 0.001 ^a^
Catechin	0.33 ± 0.002 ^b^	0.33 ± 0.001 ^a^
Epicatechin	0.23 ± 0.001 ^b^	0.25 ± 0.001 ^a^
Neochlorogenic acid	0.13 ± 0.002 ^a^	0.16 ± 0.001 ^b^
3,4*-*dihydroxy benzoic acid	0.20 ± 0.001 ^a^	0.51 ± 0.001 ^b^
p*-*Coumaric acid	0.05 ± 0.001	nd
Ferulic acid	0.07 ± 0.001 ^a^	0.16 ± 0.001 ^b^
Gallic acid	0.12 ± 0.002 ^a^	0.29 ± 0.002 ^b^
Rosmarinic acid	0.05 ± 0.001 ^a^	0.13 ± 0.001 ^b^
Cinnamic acid	0.02 ± 0.001	nd

nd—not determined; Data are expressed as mean ± SD (*n* = 3); ^a,b^—different letters indicate significantly different values in the raws (Student’s *T* test; value of *p* < 0.05 indicated statistical significance).

**Table 3:** Polar volatile metabolites in 70% ethanol extracts of hexane extracted rose flowers (HERF) and steam distilled lavender straw (SDLS), % of TIC (total ion current).

Compound	RI	HERF	SDLS
α*-*Pinene	940	0.87 ± 0.08 ^a^	0.25 ± 0.06 ^b^
β*-*Pinene	980	0.61 ± 0.05 ^a^	1.54 ± 0.08 ^b^
β*-*Myrcene	991	0.34 ± 0.07 ^a^	1.19 ± 0.09 ^b^
p*-*Cymene	1019	*-*	0.54 ± 0.07
Limonene	1025	*-*	3.55 ± 0.15
Eucalyptol	1031	*-*	3.18 ± 0.16
cis*-*beta*-*Ocimene	1040	*-*	5.41 ± 0.21
trans*-*beta*-*Ocimene	1050	*-*	3.37 ± 0.19
γ*-*Terpinene	1062	0.91 ± 0.08 ^a^	0.38 ± 0.06 ^b^
cis*-*Linalool oxide	1073	*-*	0.19 ± 0.05
trans*-*Linalool oxide	1078	*-*	0.29 ± 0.05
Terpinene	1087	2.00 ± 0.10	*-*
β*-*Linalool	1097	3.84 ± 0.10 ^a^	18.91 ± 0.15 ^b^
Phenethyl alcohol	1110	21.07 ± 0.17	*-*
cis*-*Rose oxide	1112	0.45 ± 0.05	*-*
trans*-*Rose oxide	1127	0.25 ± 0.04	*-*
Camphor	1146	*-*	0.48 ± 0.07
Borneol	1169	*-*	0.58 ± 0.10
Lavandulol	1171	*-*	6.12 ± 0.21
Terpin*-*4*-*ol	1178	0.81 ± 0.07 ^a^	3.10 ± 0.11 ^b^
α*-*Terpineol	1189	0.51 ± 0.05 ^a^	3.13 ± 0.09 ^b^
β*-*Citronellol	1228	11.26 ± 0.18	*-*
Nerol	1230	3.78 ± 0.15	*-*
Geraniol	1255	8.24 ± 0.21 ^a^	0.28 ± 0.10 ^b^
Linalyl acetate, dihydro*-*	1275	*-*	18.14 ± 0.16
(±)*-*Lavandulyl acetate	1290	*-*	4.93 ± 0.13
Citronellyl acetate	1354	0.19 ± 0.05	*-*
Eugenol	1356	2.84 ± 0.09	*-*
Neryl acetate	1364	2.09 ± 0.08 ^a^	0.95 ± 0.08 ^b^
Geranyl acetate	1383	0.63 ± 0.07 ^a^	2.94 ± 0.11 ^b^
β*-*Bourbonene	1384	3.16 ± 0.18 ^a^	0.20 ± 0.09 ^b^
β*-*Cubebene	1389	5.90 ± 0.15	*-*
β*-*Elemene	1390	0.51 ± 0.04	*-*
Methyl eugenol	1401	0.44 ± 0.05	*-*
β*-*Caryophyllene	1419	1.58 ± 0.12 ^a^	7.20 ± 0.18 ^b^
α*-*Humulene (α*-*Caryophyllene)	1454	0.34 ± 0.05 ^a^	5.06 ± 0.12 ^b^
Germacrene D	1479	0.39 ± 0.06 ^a^	2.76 ± 0.09 ^b^
α*-*Farnesene	1508	0.56 ± 0.05 ^a^	0.27 ± 0.04 ^b^
β*-*Bisabolene	1510	0.18 ± 0.04 ^a^	0.20 ± 0.03 ^a^
trans*-*Nerolidol	1564	2.60 ± 0.10 ^a^	0.27 ± 0.08 ^b^
Spathulenol	1575	1.63 ± 0.12 ^a^	0.19 ± 0.07 ^b^
Caryophyllene oxide	1580	0.32 ± 0.05 ^a^	0.30 ± 0.04 ^a^
γ*-*Eudesmol	1631	0.30 ± 0.04 ^a^	0.42 ± 0.05 ^a^
β*-*Eudesmol	1649	0.25 ± 0.05 ^a^	0.22 ± 0.03 ^a^
α*-*Eudesmol	1651	0.89 ± 0.03 ^a^	0.34 ± 0.05 ^b^
Farnesol	1714	0.34 ± 0.04 ^a^	0.55 ± 0.07 ^a^
n*-*Nonadecane	1901	4.29 ± 0.18 ^a^	0.17 ± 0.04 ^b^
n*-*Eicosane	2000	3.83 ± 0.15 ^a^	0.32 ± 0.08 ^b^
n*-*Heneicosane	2100	0.27 ± 0.06 ^a^	0.34 ± 0.08 ^a^
n*-*Docosane	2200	0.75 ± 0.08	*-*
n*-*Tricosane	2300	4.46 ± 0.21	*-*
n*-*Tetracosane	2400	1.60 ± 0.12	*-*
n*-*Pentacosane	2500	1.41 ± 0.12	*-*
n*-*Hexacosane	2600	1.41 ± 0.11	*-*

RI*—*retention index; Data are expressed as mean ± SD (*n* = 3); ^a,b^*—*different letters indicate significantly different values in the raws (Student’s *T* test; value of *p* < 0.05 indicated statistical significance).

**Table 4:** Bands assignment for the FTIR characterization of hexane extracted rose flowers (HERF), steam distilled lavender straw (SDLS) and mycelium HERF/SDLS*-*based bio*-*composite.

Assignment	Mycelium Component (Main Contribution)	HERF	SDLS	Mycelium HERF*-*Based Bio*-*Composite	Mycelium SDLS*-*Based Bio*-*Composite
		Vibration Frequency, cm^−1^			
O*-*H stretching vibration of intra and inter hydrogen bond	Polysaccharides	3429	3409	3425	3418
C*-*H stretches in methyl and methylene groups CH_2_, asymmetric stretching	Lipids, Polysaccharides	2966	2927	2926	2927
CH_2_ symmetric stretching	Lipids, Polysaccharides	2854	2855	2854	2854
C=O stretching in xylans (hemicellulose), pectins; ester bonds	Lipids, Polysaccharides	1737	1733	1732	1728
(amide I in β *-*sheets secondary structures) Absorbed O*-*H associated with lignin or cellulose; a characteristic band for cis*-* HRC = CR’H bonds,	Proteins; Lipids, Polysaccharides	1652; 1620	1630	1665 1643	1643
Amide II	Proteins	*-*	*-*	1548	1548
a characteristic band of the type C=C; C=C stretching of aromatic ring (syringyl) in lignin C=C stretcing of aromatic ring (guaiacyl) in lignin C=C stretching of aromatic ring (syringyl) in lignin	Polysaccharides	1516	1516	1537;1548	1537;1548
νC*-*Hs(CH_2_) in pyranise ring,	Polysaccharides	1454	1432	*-*	1454;1462
C*-*H bending in chitin, cellulose and hemicellulose	Polysaccharides	1370	1377	1377	1378
CH_2_ wagging in cellulose	Polysaccharides	1318	1318	*-*	*-*
PO_2_*-* asymmetric stretching, C*-*O stretching in lignin and xylan, Nucleic acids	Nucleic acids Polysaccharides	1239	1246	1254	1250
1C*-*O*-*C vibration in cellulose and hemicellulose	Polysaccharides	1150	1161	1152	1161
		*-*	1114	*-*	1110
C*-*O valence vibration from C3*-*O3H	Polysaccharides	1057	1055	1046	1047
C*-*O stretching in cellulose	Polysaccharides	1053	1055	1055	1047
C*-*C stretching	Polysaccharides	1034	1055	1046	1047
β(COH), β(CH) of C*-*1, νs(COC) in glycosidic linkage, ring modes	Polysaccharides	928	916	916	919
Anomer C*-*group, Glucan β*-*anomer C*-*H bending, C*-*H deformation in cellulose, ν(CC), β(CCH)	Polysaccharides	899	878	855	842
CH_2_*-*rocking	Polysaccharides	761	*-*	769	*-*

**Table 5:** Physical and mechanical properties of the mycelium hexane extracted rose flowers (HERF)/ steam distilled lavender straw (SDLS)*-*based bio*-*composites.

Mycelium Bio*-*Composite	Apparent Densityρ_a_	Capillary AbsorptionWc	Water Absorption				Compressive Resistance at 10% Deformation σ_10_
			1 Day		28 Days		
			W^v^_a,1d_	W^m^_a,1d_	W^v^_a,28d_	W^m^_a,28d_	
	kg/m^3^	kg/m^2^	%vol.	%wt.	%vol.	%wt.	kPa
HERF	462 ± 10.2	3.4 ± 0.38	20.3 ± 3.45	43.9 ± 7.5	58.2 ± 7.2	126.0 ± 15.7	1029 ± 51
SDLS	347 ± 3.7	6.5 ± 0.20	39.8 ± 5.72	114.6 ± 14.5	85.0 ± 3.5	245.0 ± 10.1	718 ± 22

The standard deviation is determined with triplicate samples (mean ±standard deviation).

## Data Availability

The raw/processed data required to reproduce these findings cannot be shared at this time as the data also forms part of an ongoing study.
